# Supplementary material for: Empirical methods for controlling false positives and estimating confidence in ChIP-Seq peaks
Source: BMC Bioinformatics. 2008 Dec 5;9:523. doi: 10.1186/1471-2105-9-523 (PMC2628906; doi:10.1186/1471-2105-9-523)
Supplement: Additional File 1 — A variety of html documents from the USeq web site detailing the available applications, their best usage, output file type descriptions, command line menus, etc. [file 1471-2105-9-523-S1.zip › USeqUserGuides/JavaDoc.zip!/JavaDoc/util/apps/package-tree.html]

util.apps Class Hierarchy


|  |  |  |
| --- | --- | --- |
| **Overview** \| **Package** \| Class \| Use \| **Tree** \| **Deprecated** \| **Index** \| **Help** | |  |
| **PREV**   **NEXT** | **FRAMES**    **NO FRAMES**     **All Classes** |  |


---


## Hierarchy For Package util.apps

**Package Hierarchies:**: All Packages

---

## Class Hierarchy

- java.lang.Object
  - util.apps.**ArrayPrinter**- java.awt.Component (implements java.awt.image.ImageObserver, java.awt.MenuContainer, java.io.Serializable)
      - java.awt.Container
        - javax.swing.JComponent (implements java.io.Serializable)
          - javax.swing.JPanel (implements javax.accessibility.Accessible)
            - util.apps.**ScatterDrawPanel**
          - java.awt.Window (implements javax.accessibility.Accessible)
            - java.awt.Frame (implements java.awt.MenuContainer)
              - javax.swing.JFrame (implements javax.accessibility.Accessible, javax.swing.RootPaneContainer, javax.swing.WindowConstants)
                - util.apps.**ScatterDrawFrame**
      - util.apps.**Correlate**- util.apps.**Delme2**- util.apps.**FileJoiner**- util.apps.**FileSplitter**- util.apps.**FileTrimmer**- util.apps.**FilterAgilentProbeFile**- util.apps.**Flash**- util.apps.**GeneCentricCNVParser**- util.apps.**GeneNameCounter**- util.apps.**HouseBean**- util.apps.**IntersectLists**- util.apps.**JQSub**- util.apps.**MakeBioTools**- util.apps.**MergeColumns**- util.apps.**ParseMLSHTML**- util.apps.**ParserForGff**- util.apps.**PrintSelectColumns**- util.apps.**RenameRWSResults**- util.apps.**ScatterPlot**- util.apps.**ScoreChromosomes**- util.apps.**ScoreSequences**- util.apps.**SmoothData**- util.apps.**StatBarFiles**- util.apps.**SubtractOneFromEachColumn**- util.apps.**TASSignalChromFileSplitter**- util.apps.**TextParser**- util.apps.**WindowScoreSmoother**- util.apps.**ZipAndDelete**

---


|  |  |  |
| --- | --- | --- |
| **Overview** \| **Package** \| Class \| Use \| **Tree** \| **Deprecated** \| **Index** \| **Help** | |  |
| **PREV**   **NEXT** | **FRAMES**    **NO FRAMES**     **All Classes** |  |


---
